# Mutation analysis of the *TUBB8* gene in primary infertile women with oocyte maturation arrest

**DOI:** 10.1186/s13048-022-00971-9

**Published:** 2022-03-30

**Authors:** Zhongyuan Yao, Jun Zeng, Huimin Zhu, Jing Zhao, Xiaoxia Wang, Qiuping Xia, Yanping Li, Lingqian Wu

**Affiliations:** 1 Reproductive Medicine Center, Xiangya Hospital, Central South University, Changsha, 410078 Hunan China; 2 Center for Medical Genetics & Hunan Key Laboratory of Medical Genetics, School of Life Sciences, Central South University, Changsha, 410078 Hunan China; 3 Clinical Research Center for Women’s, Reproductive Health in Hunan Province, Hunan, China

**Keywords:** Oocyte MI arrest, Female infertility, *TUBB8*, Mutation

## Abstract

**Background:**

Oocyte maturation arrest at metaphase I leads to fertilization failure in humans. In early embryos, the tubulin beta 8 class VIII (*TUBB8*) encodes a β-tubulin isotype and aids in the assembling of the human oocyte spindle. Mutations in the *TUBB8* potentially interfere with human oocyte maturation—a crucial prerequisite for fertilization and subsequent embryonic development. This study aims to investigate the novel mutations in *TUBB8* and their prevalence.

**Results:**

Hundred fertile women (controls) and eleven infertile women with oocyte maturation arrest were chosen for the study. A total of five *TUBB8* heterozygous/homozygous mutations were found in eleven infertile females (p.A313V, p.C239W, p.R251Q, p.P358L, and p.G96R). The Exome Aggregation Consortium (ExAC), SIFT, and PolyPhen-2 analyses revealed that p. A313V has unknown pathogenicity and p.C239W, p.R251Q, p.P358L, and p.G96R have possible pathogenicity. The wild-type (WT) and four mutant gene constructs were transfected to Hela cells. The Western blot analysis indicates that the *TUBB8* expression of the p.C239W, p.R251Q, and p.G96R mutations was significantly decreased than that of WT. The immunofluorescence assay showed that the Hela cells transfected with either p.C239W, p.R251Q, or p.G96R mutations exhibited the disrupted microtubule structure, revealing a significant difference in the organization of the microtubule network compared to the WT.

**Conclusions:**

We identified three novel variants and two reported variants out of 11 infertile women with oocyte metaphase I arrest. According to the present data, *TUBB8* gene variants account for 31.96% of all participants (109/341) with oocyte maturation arrest.

**Supplementary Information:**

The online version contains supplementary material available at 10.1186/s13048-022-00971-9.

## Background

Oocyte maturation is a lengthy process in humans, especially during in vitro fertilization (IVF) where it can be impeded at any stage, such as the germinal vesicle (GV) stage, the metaphase I (MI) stage, or the metaphase II (MII) stage, resulting in fertilization failure. The first case of maturation failure of a human oocyte was reported in 1990 [1]. However, few cases of primary infertility with oocyte maturation arrest have been reported [2–6]. Oocyte maturation begins with a surge of luteinizing hormones and a breakdown of GV, resuming meiosis, followed by spindle assembly, chromosome migration, asymmetric division, completion of the first meiotic division, and finally, the discharge of the first polar body [7, 8]. MII stage oocytes can only successfully fertilize and form a zygote by fusing with a sperm cell [8, 9]. The spindle assembly and chromosome separation are error-prone in humans, leading to aneuploidy and even oocyte maturation arrest [10, 11]. A defect in spindle formation can lead to MI arrest during meiosis. Meiotic spindles consist of microtubules which are dynamic polymers composed of alpha/beta-tubulin isomers [12, 13]. Multiple genes encode β-tubulin, including nine subtypes: *TUBB1*, *TUBB2A*, *TUBB2B*, *TUBB3*, *TUBB4A*, *TUBB4B*, *TUBB5*, *TUBB6*, and *TUBB8*. Tubulins rely on cell-specific subtype’s expression and possess special post-translational modification-dependent cellular functions [14]. TUBB8 is the major β-tubulin isotype that occupies most of the expressed β-tubulin in early embryos and participates in the human oocyte spindle assembling [15].

Several mutations of *TUBB8* have been reported, suggesting that *TUBB8* encodes a β-tubulin isotype of previously undetermined function [15, 16]. Those studies disclosed that a heterozygous or homozygous mutation in the *TUBB8* causes oocyte maturation arrest in infertile women. These mutations, either inherited paternally in an autosomal dominant fashion or arising de novo [17], interfere with the maturation of human oocytes, fertilization or pre-implantation embryonic development, and even embryo implantation failure. In addition, several studies in recent years have extended the spectrum of *TUBB8* mutations. Some mutations are associated with new phenotypes, such as multiple pronuclei (MPN) in zygotes after IVF or intracytoplasmic sperm injection [18]. These findings uncovered an essential role for *TUBB8* in human oocyte maturation and female fertility, and enhanced the understanding of how *TUBB8* mutations interfere with zygote development. *In-vitro* testing of *TUBB8* mutations revealed that *TUBB8* dominantly exerts a negative effect by preventing the oocyte maturation through disruption of microtubule formation, meiotic spindle assembly, and microtubule dynamics [17, 19–24]. Identifying additional novel variants and new phenotypes caused by variants is essential because it will provide the foundation for exploring the comprehensive effects of *TUBB8* in the early human reproductive process.

In the present study, we focused on continuously expanding the sample size to detect the mutation frequency of the *TUBB8* gene during oocyte maturation arrest in infertile women, emphasizing the prevalence of new *TUBB8* mutations during that process. We discovered that three novel *TUBB8* mutations extended the dysfunctional phenotype originally caused by *TUBB8* mutations.

## Methods

### Selection of participants

Eleven patients with total fertilization failure caused by abnormal development of oocytes and embryos from ten independent families were recruited for the study. Each family (referred by Xiangya Hospital) had one or more female members with a history of persistent infertility. All patients had a normal karyotype (46, XX), and DNA samples were obtained from all of them. We also extracted samples from 100 unrelated, anonymous and childbearing women (as control) for this study.

### Sampling and DNA isolation

Peripheral blood samples from patients and their families were collected, and genomic DNA samples from peripheral blood were obtained using the Quick Gene DNA whole blood kit L (Quick Gene, Holliston, United States) according to the manufacturer’s protocol.

The coding regions of *TUBB8* were amplified using specific primers (Table 1), and the amplicons were sequenced using Sanger sequencing—ABI 3100 DNA analyzer (Applied Biosystems).

**Table 1:** Amplification primers of TUBB8

Exon	F/R^a^	Amplification primers	PCR size (bp)
**1–3**	F	CGGGGCTATTTAAACGTTGG	805
	R	CCCAGAGGATGACCTTAGCA	
**4**	F	GTGTGACGCTTGGCTCTTTC	1266
	R	TTAAAACGCAGCAGGAGATG	

*BP* Base pair

^a^F represents forward primers and R represents reverse primers

### Detection of mutations

After getting the sequenced data, mutation annotation was done using the following databases: ExAC, dbSNP, SIFT, and PolyPhen 2. The protein 3D locations of the disease-associated variants were viewed via the *SWISS-MODEL* program.

### Quantitative analysis of *TUBB8* mutations in cultured Cells

A full-length TUBB8 cDNA cloned in a pCDNA3.1 vector with a CMV promoter and an in-frame C-terminal FLAG tag was purchased from OriGene, Inc. Point mutations were generated by quick-change polymerase chain reaction for the expression of the wild-type (WT) and four mutations (except for p.P358L, which was reported previously). The pCDNA3.1 vector was transfected into Hela cells using Lipofectamine 2000 (Thermo Fisher Scientific, Waltham, MA, USA). Forty-eight hours after transfection, cultured Hela cells were dissolved in RIPA lysis buffer (Beyotime, China) and proteinase inhibitor. The protein expression was detected by Western blot.

### Expression of WT and mutant forms of *TUBB8* in cultured Cells

After 48 h of transfection, the cells were fixed, permeabilized, stained, and analyzed using confocal laser-scanning microscopy (Leica). The cells were labeled with DAPI (to visualize nucleus), FLAG (to visualize transgene), and α-tubulin (to visualize endogenous microtubule network).

## Results

**Fig. 1:**
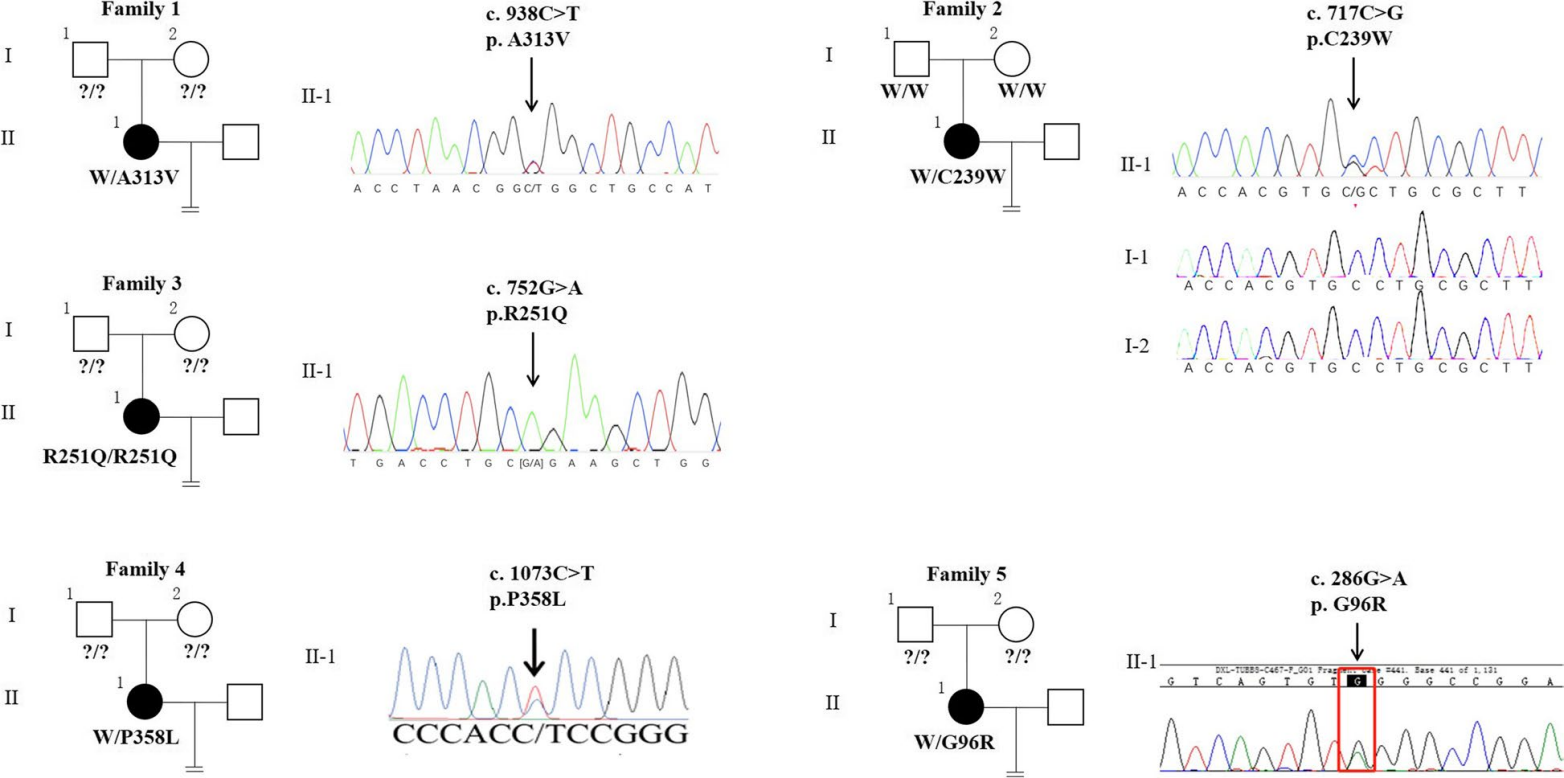
Pedigrees of five families with mutations in *TUBB8*. Sanger sequencing chromatograms are shown to the right of the pedigrees. The “ = ” sign indicates infertility. Black circles represent affected individuals, and question marks indicate the absence of DNA samples

**Fig. 2:**
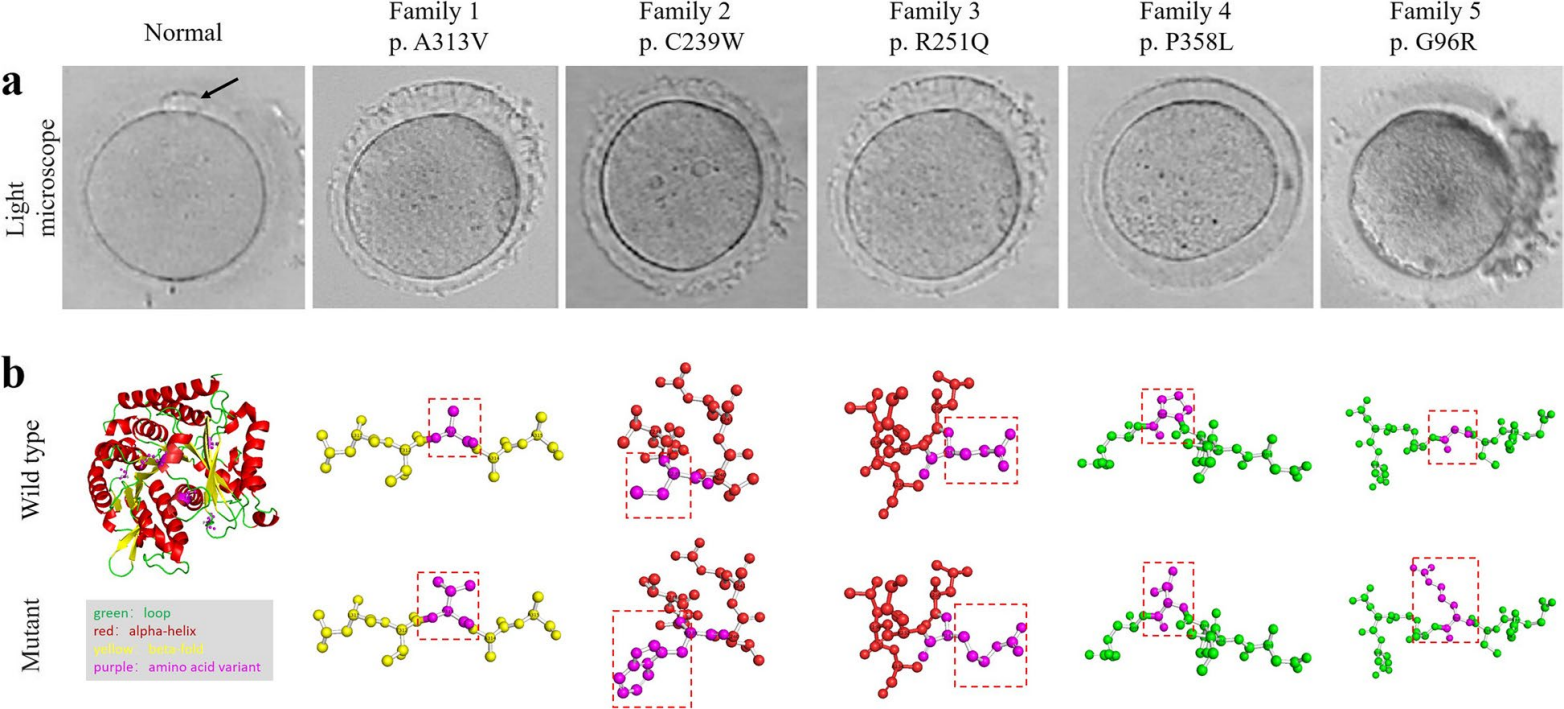
Phenotypes of oocytes from patients with mutations of *TUBB8*. **a** The morphologies of the MI or MII oocytes examined by light microscopy. The arrow represents the polar body. **b** The protein 3D structure of the disease associated variants rendered by *SWISS-MODEL* program. The variation of p.A313V was polar, neutral amino acid in the β-fold region, while p.C239W occurred in the α-helix region. The variation of p.R251Q changed from negatively charged amino acid to positively charged amino acid, which affected the formation of hydrogen bond in the secondary structure of protein and may affect the structural stability of protein. p.P358L was nonpolar amino acid. The variation of p.G96R changed from polar, neutral to positively charged amino acid

All patients had experienced primary infertility in the past 1–6 years, with each family having one or more female members with a history of persistent infertility. Their spouses had normal sperm count, morphology, and motility. Each patient had experienced 1–3 cycles of *in-vitro* fertilization failure (Table 2). A total of five heterozygous/homozygous mutations in *TUBB8* were found (Fig. 1). Patient II-1 from family 1 had been diagnosed with primary infertility for 6 years at 33 years. Although 8 oocytes were obtained, only 1 was matured and fertilized, whereas the others were arrested at meiosis I. For family 2, 16 oocytes were retrieved from two ICSI cycles, and only 1 oocyte was fertilized. For families 3 and 5, the patients underwent two or three IVF and ICSI cycles; all oocytes retrieved were in MI. Though the patient from family 4 had only been infertile for 1 year and had experienced 1 IVF cycle, of the 14 aspired oocytes, 10 arrested at the MI stage, and 4 oocytes were still in the GV stage after being cultured for 72 h in a medium (Fig. 2a). The genetic analysis was done and five missense *TUBB8* mutations (c. 938C > T [p. A313V], c.717C > G [p.C239W], c.752G > A [p.R251Q], c.1073C > T [p.P358L], and c.286G > A [p.G96R]) were later discovered. None of the *TUBB8* gene mutations were found in additional six patients with primary infertility from five different families.

**Table 2:** Clinical characteristics of oocyte maturation arrest from the affected patients

Case	*TUBB8* mutation	Age (years)	Duration of infertility (years)	Previous IVF/ICSI cycles	Total no. of oocytes retrieved	Stage or Stages of Oocytes
Family 1, Patient II-1	c. 938C > T (p. A313V)	33	6	1	8	7 in MI, 1 with abnormal morphologic features
Family2, Patient II-1	c.717C > G (p.C239W)	32	5	2	16	15 in MI, 1 with abnormal morphologic features
Family 3, Patient II-1	c.752G > A (p.R251Q)	30	3	3	20	20 in MI
Family 4, Patient II-1	c.1073C > T(P.P358L)	27	1	1	14	10 in MI, 4 in GV
Family 5, Patient II-1	c.286G > A (p.G96R)	33	8	2	21	21 in MI

We have also sequenced 100 unrelated childbearing women with the *TUBB8* gene by Sanger to exclude the possibility that the newly discovered mutations might not only be prevalent in the study population. It was revealed that the above five mutations were not found in the control DNA samples and the dbSNP (v142). Furthermore, the ExAC browser analysis revealed that c.938C > T had a low allele frequency (3/116668), even though the p.A313V is relatively variable in mammals. Analysis with MultAlin (Supplementary Fig. 1) showed that amino acids are highly conserved in positions p.C239W, p.R251Q, p.P358L and p.G96R. Additionally, SIFT and PolyPhen 2 predicted that these four variants would be detrimental to TUBB8 protein function (Table 3), while p.A313V seemed to be benign.

**Table 3:** Effects of TUBB8 mutations predicted with in-silico tools

cDNA alteration	Amino acid alteration	Exon	Frequency in our cohort	dbSNP	ExAC allele frequency	ExAC homozygotes frequency	SIFT^a^	PolyPhen 2^b^
c. 938C > T	p. A313V	4	1/11	Not found	3/116668	Not found	N	0.013(B)
c.717C > G	p.C239W	4	1/11	Not found	Not found	Not found	D	0.999(D)
c.752G > A	p.R251Q	4	1/11	Not found	Not found	Not found	D	0.966(D)
c.1073C > T	p.P358L	4	1/11	Not found	Not found	Not found	D	1.0(D)
c.286G > A	p.G96R	4	1/11	Not found	Not found	Not found	D	1.0(D)

^a^Effects of mutation predicted by SIFT

^b^Effects of mutation predicted by Polyphen 2

*B* Benign, *D* Deleterious, *N* Neutral

The homology modeling of the disease-associated variants was performed using the WT proteins whose experimentally validated structure was available on Protein Data Bank as the model, and it was displayed as the most informative protein 3D structure using the *SWISS-MODEL* program (Fig. 2b) [25]. The mutation p.A313V has occurred in the β-fold region, while p.C239W and p.R251Q were found in the α-helix region. The main form of protein secondary structure is the α-helix. The composition and sequence of amino acids in α-helix significantly affect its formation and stability. β-folding is widespread in the secondary structure of proteins and without like charges, which is beneficial to the extension of polypeptide chains. Other mutations such as p.G96R and p.P358L occur in the loop region, primarily located on the surface of protein molecules and used as protein binding and enzyme catalytic sites. The changes of the functional domains further supplemented that p. A313V seemed to be benign, while in contrast, the other four variants would be harmful to TUBB8 protein function.

**Fig. 3:**
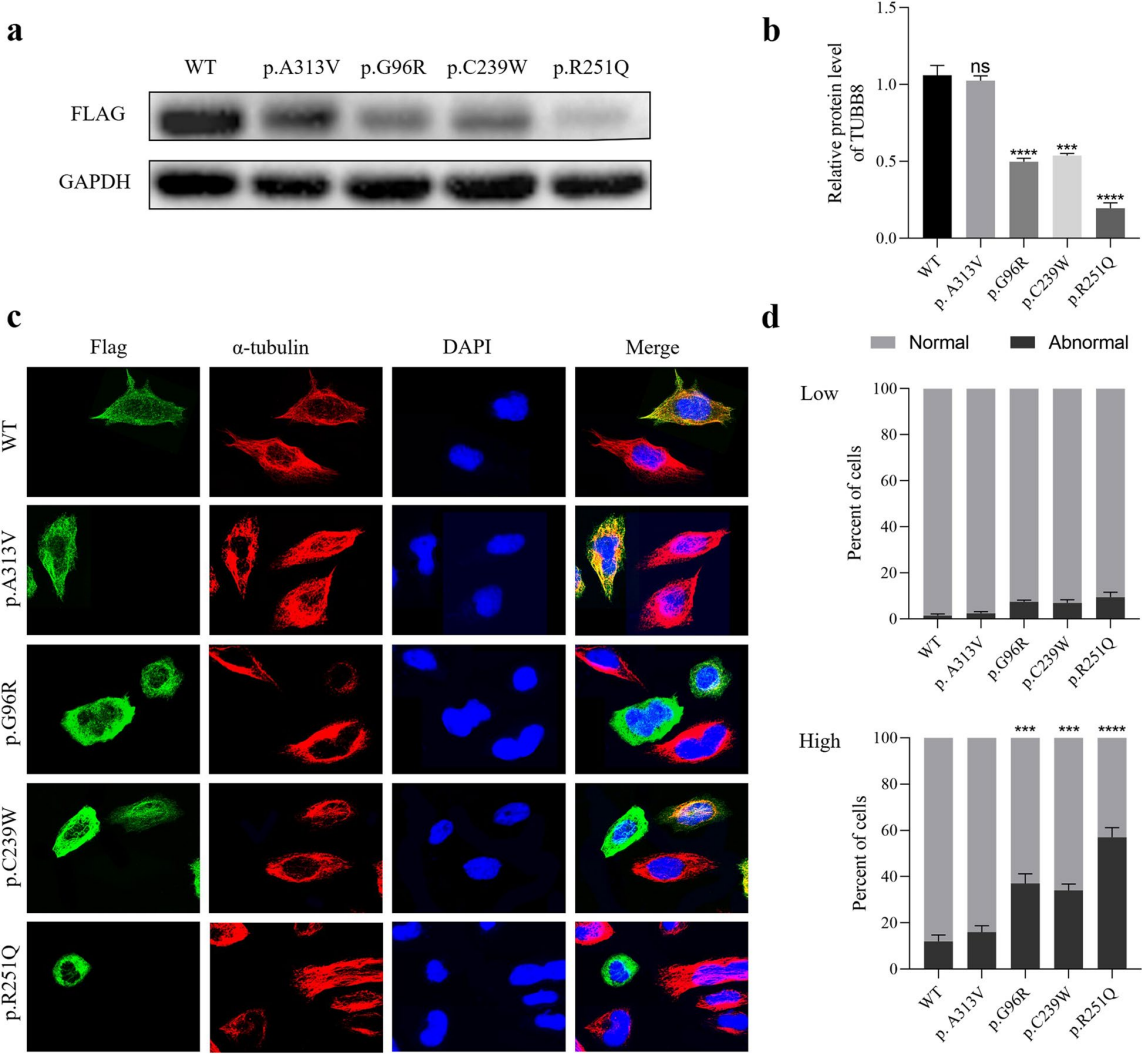
*TUBB8* expression analysis in cultured cells. **a-b** The expression level of WT, p.A313V, p.G96R, p.C239W, and p.R251Q *TUBB8* protein in Hela cells detected by Western Blot. **c-d** Microtubule morphology and phenotypes of Hela cells transfected with constructs engineered to express C-terminally FLAG-tagged *TUBB8* (WT and mutants) and examined by immunofluorescence microscopy. The cells were stained with DAPI to visualize nucleus (blue), FLAG to visualize transgene (green) and α-tubulin to visualize endogenous microtubule network (red)

In transfected Hela cells, the Western blot analysis indicates that the *TUBB8* expression of p.A313V mutation showed no significant influence while p.C239W, p.R251Q, and p.G96R mutations significantly decreased than that of WT (Fig. 3a, b). Immunofluorescence assay revealed that when the exogenous TUBB8 proteins were expressed at a relatively low level, the WT and mutant proteins could co-assemble into a microtubule network without triggering severe structural damage (Fig. 3d). In contrast, high levels of mutant TUBB8 expression resulted in significantly higher abnormal rates than WT variant (Fig. 3c, d) (*P* < 0.01). Moreover, the p.A313V mutation showed a comparable abnormal rate with WT. The expression trend was in accordance with the prediction.

## Discussion

The prevalence of *TUBB8* mutations in 11 infertile women with oocyte maturation defects was established in this study by genetic analysis. We found three novel variants (c.717C > G; (p.C239W), c.752G > A; (p.R251Q), and c.286G > A; (p.G96R)) and two previously reported variants (c. 938C > T; (p. A313V) and c.1073C > T; (p.P358L)) in *TUBB8* from 10 independent families with infertile females. One of them is homozygous mutations (c.752G > A; (p.R251Q)). Except for p.P358L mutation, the p.C239W, p.R251Q, and p.G96R mutations are predicted to be highly pathogenic *in-silico* and *in-vitro* assays. It may significantly expand the phenotype of *TUBB8* mutation-induced oocyte dysfunction in humans.

Almost all previous research has shown that heterozygous *TUBB8* missense mutations cause oocyte maturation arrest via dominant-negative effects. However, in a consanguineous mating family where the proband underwent primary infertility, a novel *TUBB8* variant was discovered [26]. The patient with homozygous p.A54V *TUBB8* was infertile, but her parents were not affected by the heterozygous p.A54V missense mutations. These findings suggest that p.A54V has a haploinsufficiency effect than a dominant-negative effect. Our study found a similar case in a patient with homozygous p.R251Q mutation while her mother was fertile. We speculate that the p.R251Q heterozygous mutation would have no effect on female fertility as the proband’s mother was a carrier. As a result, different effects could explain why different *TUBB8* mutations result in diverse protein structural defects and further change the protein interactions with kinesins or binding to other microtubule-related proteins.

Of the 11 individuals from ten different families, 5 (45.5%) were genetically diagnosed with *TUBB8* heterozygous/homozygous mutations. According to the existing data, the variants of the *TUBB8* gene account for 31.96% of all participants (109/341). A few recurrent mutations were found in these studies, including c.10A > C, c.292G > A, c.527C > T, and c.763G > A, suggesting that these *TUBB8* mutations have a higher incidence in patients with oocyte maturation disorders [15–17, 22, 26–28]. *TUBB8* mutations have been linked to phenotypic variability, according to recent research. To date, a total of 109 unique *TUBB8* variants were reported, including 87 heterozygous mutations, 13 homozygous mutations, and compound heterozygous variants, present in 8 families (Supplementary Table 1). According to these reports, *TUBB8* mutations account for about 31.96% of all cases of primary oocyte maturation arrest; nevertheless, other genetic causes of MI and GV blocks remain unclear. These mutations may influence microtubule formation by affecting: the stability of β-tubulin, the lateral contacts between the precursors (protofilaments), or the production of material migrating as a native heterodimer, altering the microtubule tissue expression and causing oocyte MI arrest [29]. These findings imply that diverse *TUBB8* mutations may lead to complete stagnation of oocyte/embryonic phenotypic variability in GV and MI phases. Because oocyte maturation proceeds in different stages, the genetic factors that cause female infertility, including those associated with GV and MI phases, remain largely unknown. Therefore, depending on the oocyte maturation stage, additional unknown genetic defects may also cause infertility. Several studies have been published describing infertility cases with oocyte maturation arrest; however, heterozygous *TUBB8* mutations were not discovered during our work on the genetic basis of oocyte arrest until 2016. As of today, some *in-vivo* studies have been exploring the treatment for infertile patients with *TUBB8* mutations [30]. By injecting WT *TUBB8* cRNA into mouse oocytes—where the mutant *TUBB8* was expressed—the blastocyst rate was significantly improved and finally acquired normal mouse offspring. This finding indicates that supplementing with exogenous WT *TUBB8* could improve mutant *TUBB8-*induced aberrant phenotypes and provide a theoretical basis for treating patients who are infertile due to *TUBB8* mutations.

In recent years, there have been more reports of oocyte arrest disorders caused by mutations in *TUBB8* and newly associated genes. It has been shown that *PATL2* [31–33] plays an essential role in maintaining the integrity of a small fraction of synthesized mRNAs during oocyte growth, which is a necessary step in post-fertilization and early embryonic development. Mutations in *TRIP13*, a critical component of the spindle assembly checkpoint, are reported to cause oocyte maturation arrest or abnormal zygote cleavage [34]. These genes play a vital role in oocyte growth and maturation by influencing or regulating the expression of crucial protein-encoding mRNAs in the meiotic process. A mutation that affects the chaperone-dependent folding or assembly of α/β-tubulin heterodimer could disrupt microtubule formation resulting in oocyte meiotic deficiency in humans. These findings reckon that oocyte maturation arrest could be caused by other unknown genetic defects that are yet to be identified.

At present, primary infertility is associated with oocyte maturation arrest necessitating donor eggs for successful reproduction. However, the problem of oocyte maturation stagnation still needs to be identified and handled. We believe that identifying it would be highly beneficial in future cases of primary infertility.

## Conclusions

We discovered that three novel *TUBB8* mutations extended the dysfunctional phenotype initially caused by *TUBB8* mutations. Thus, according to the previous studies, *TUBB8* variants accounted for 31.96% (109/341) of patients with an oocyte or embryonic abnormalities, indicating that these defects are likely related to TUBB8 variants with high genetic variability and phenotypic diversity. TUBB8 should be considered a genetic marker for genetic counseling in infertile women with oocyte or embryonic defects to improve the efficiency of genetic diagnosis.

## Supplementary Information

**Additional file 1:** **Supplementary Figure 1.** Analysis with MultAlin of TUBB8. High consensus is represented in red colour and low consensus is represented in blue or black colour. The black arrow labeled the variants identified.

**Additional file 2:** **Supplementary Table 1.** Summary of reported TUBB8 mutations (see Supplementary Table 1.xlsx).

## Data Availability

All data generated or analysed during this study are included in this published article (and its supplementary information files).

## Abbreviations

*TUBB8*: Tubulin beta eight class VIII
IVF: In vitro fertilization
GV: Germinal vesicle
MI: Metaphase I
MII: Metaphase II
