# Supplementary figures and images for: Mutation analysis of the TUBB8 gene in primary infertile women with oocyte maturation arrest
Source: J Ovarian Res. 2022 Mar 30;15:38. doi: 10.1186/s13048-022-00971-9 (PMC8969352; doi:10.1186/s13048-022-00971-9)

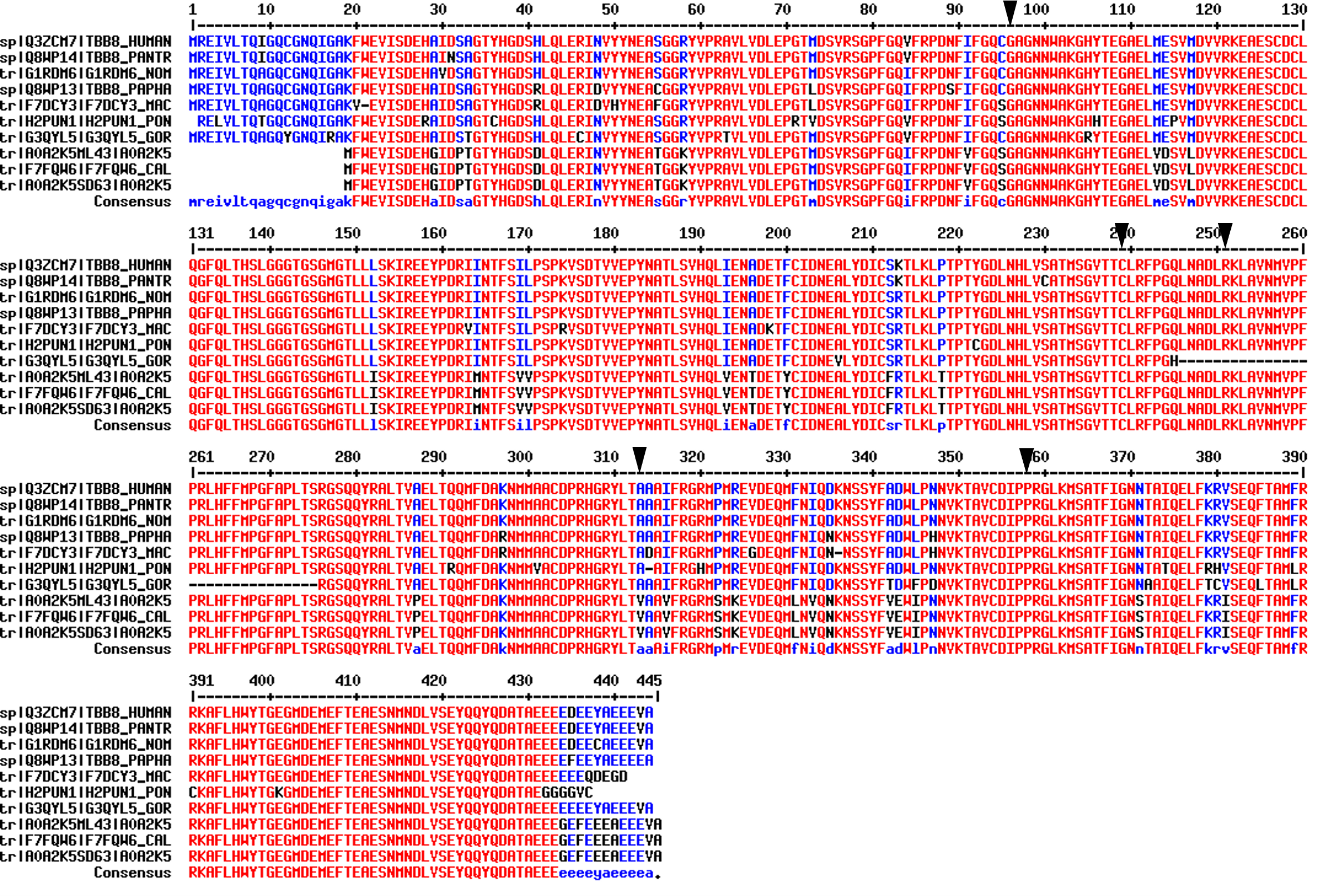

Supplement: Supplementary file 1 — Additional file 1: Supplementary Figure 1. Analysis with MultAlin of TUBB8. High consensus is represented in red colour and low consensus is represented in blue or black colour. The black arrow labeled the variants identified. [file 13048_2022_971_MOESM1_ESM.png]
